# *Bridge*: A New Algorithm for Rooting Orthologous Genes in Large-Scale Evolutionary Analyses

**DOI:** 10.1093/molbev/msae019

**Published:** 2024-02-02

**Authors:** Leonardo R S Campos, Sheyla Trefflich, Diego A A Morais, Danilo O Imparato, Vinicius S Chagas, Ricardo D’Oliveira Albanus, Rodrigo J S Dalmolin, Mauro A A Castro

**Affiliations:** Bioinformatics Multidisciplinary Environment–BioME, IMD, Federal University of Rio Grande do Norte, Natal, Brazil; Bioinformatics and Systems Biology Laboratory, Federal University of Paraná, Curitiba 81520-260, Brazil; Bioinformatics Multidisciplinary Environment–BioME, IMD, Federal University of Rio Grande do Norte, Natal, Brazil; Bioinformatics Multidisciplinary Environment–BioME, IMD, Federal University of Rio Grande do Norte, Natal, Brazil; Bioinformatics and Systems Biology Laboratory, Federal University of Paraná, Curitiba 81520-260, Brazil; Department of Psychiatry, Washington University School of Medicine, St. Louis, MO, USA; Bioinformatics Multidisciplinary Environment–BioME, IMD, Federal University of Rio Grande do Norte, Natal, Brazil; Department of Biochemistry, CB, Federal University of Rio Grande do Norte, Natal, Brazil; Bioinformatics and Systems Biology Laboratory, Federal University of Paraná, Curitiba 81520-260, Brazil

**Keywords:** evolutionary rooting, systems biology, systems evolution

## Abstract

Orthology information has been used for searching patterns in high-dimensional data, allowing transferring functional information between species. The key concept behind this strategy is that orthologous genes share ancestry to some extent. While reconstructing the history of a single gene is feasible with the existing computational resources, the reconstruction of entire biological systems remains challenging. In this study, we present *Bridge*, a new algorithm designed to infer the evolutionary root of orthologous genes in large-scale evolutionary analyses. The *Bridge* algorithm infers the evolutionary root of a given gene based on the distribution of its orthologs in a species tree. The *Bridge* algorithm is implemented in *R* and can be used either to assess genetic changes across the evolutionary history of orthologous groups or to infer the onset of specific traits in a biological system.

## Introduction

The process of transferring functional information between species allows experimental knowledge bases of model organisms to be used to investigate nonmodel organisms, and orthology annotation between species is essential for this process (Kumar et al. 2023). For example, the systematic discovery of essential functions of human cells at the developmental level comes mainly from mice, yeast, and worms (de Souza et al. 2021). Another direct benefit of transferring functional information is the study of poorly annotated genomes, which can be preliminarily described by comparing them with evolutionarily related organisms. Using this strategy, one can explore gene families, motifs, and protein domains (Glover et al. 2019), providing invaluable information to investigate the evolution of genetic systems, such as the origin of biochemical pathways (Mirkin et al. 2003; Castro et al. 2008; Dalmolin et al. 2011; Viscardi et al. 2021). However, the evolutionary investigation of genetic systems involving multiple genes is still challenging for the existing computational resources, especially when seeking to infer the evolutionary root of a system. For example, the *PARS* algorithm (Mirkin et al. 2003) reconstructs parsimonious evolutionary scenarios for a set of orthologs with a particular phyletic pattern in a given species tree. However, it does not provide a high-throughput solution to track the onset of a large number of genetic traits. To our knowledge, no current computational framework is available for such a task.

Here, we present *bridge*, a new algorithm designed to deal with large-scale evolutionary analyses, using orthology data to infer the most ancient genetic archetype of a given gene. The *bridge* algorithm infers the evolutionary root of a given gene based on the distribution of its orthologs in a species tree. The *bridge* algorithm is implemented in *R* and can be used to find the last common ancestor (LCA) of orthologous groups (OGs), using this information to assign evolutionary roots to genes.

## A Framework to Explore Network Evolutionary Scenarios

**Fig. 1.**
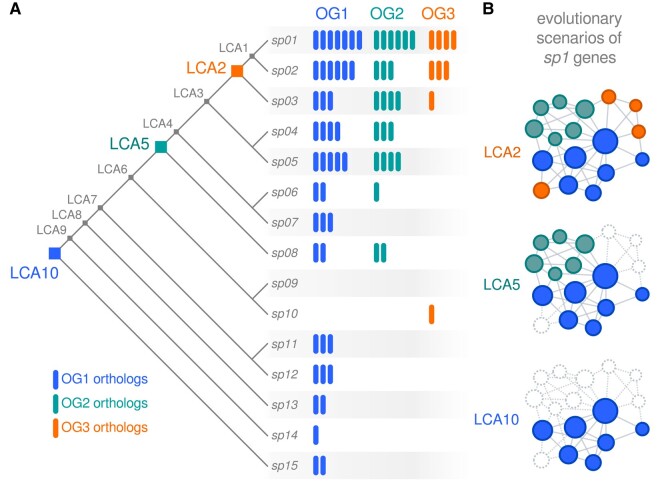
Evolutionary scenarios of genes from a hypothetical gene network. a) A species tree representing the phylogenetic relationships between species in the leaves (named from *Sp01* to *Sp15*). The colored bars represent the number of orthologous genes assigned to each species. This example illustrates the distribution of orthologs from 3 OGs (named from OG1 to OG3). The colored diamonds represent the evolutionary rooting of each OG calculated by the *bridge* algorithm. b) A hypothetical protein–protein interaction network reconstructed at different evolutionary levels by projecting the rooting information.

The evolutionary history of an OG might be the result of a complex phyletic pattern involving gene duplication, gene deletion, and horizontal gene transfer (HGT; Koonin 2005). Figure 1A exemplifies the phyletic pattern of 3 hypothetical OGs in 15 species. The OG1 has orthologs in almost all species of the hypothetical tree, except species *sp9* and *sp10*, representing a gap in the OG1's history. This gap might be explained by at least 2 scenarios: an independent emergency of OG1 in LCA10 and LCA5 or a lineage-specific gene loss in the branch involving *sp9* and *sp10*. Similarly, OG3 has orthologs in *sp1*, *sp2*, and *sp3*, and an isolated ortholog in *sp10*. The evolutionary history of the hypothetical OG3 may also be explained by gene loss and/or HGT. The *Bridge* algorithm assesses the phyletic pattern of a given OG in order to assign its evolutionary root. For OG1, a possible evolutionary scenario involves a lineage-specific gene loss, while for OG3, it involves an independent gene emergence or HGT. The inferred evolutionary root for each OG is represented by colored diamonds in the species tree (Fig. 1A).

Figure 1B illustrates a typical usage scenario, using a schematic network with the 17 genes annotated in the species *sp01*, which is regarded as the reference species in this example and for which we want to investigate the evolutionary root of its genes. The gene network represents a hypothetical genetic system described for an extant organism, and only genes (not associations between genes) are traced to the species tree. The top network shows that all *sp01* genes are represented in LCA2, implying that the LCA of *sp01*, *sp02*, and *sp03* may have had a similar genetic system. Also, 4 *sp01* genes (orange nodes) are rooted at LCA2, with all its descendants having at least one copy of the ancestral sequence. The subsequent networks query the same genetic system at other levels of the species tree: 6 *sp01* genes are rooted at LCA5 (green nodes) and 7 *sp01* genes are rooted at LCA10 (blue nodes). Note that once a gene is rooted at a given point of the species tree, then the corresponding network node is represented in all levels of the phyletic lineage. Possible questions we may ask from reconstructing a network evolutionary scenario include the following: At which levels of a phylogenetic tree do the subcomponents of a network have all the elements for the onset of a system as we understand it in an extant organism? Are there classes of network elements that may have arisen earlier in the evolutionary history of a given system? The *bridge* algorithm provides a framework to explore these questions by reconstructing network evolutionary scenarios.

## The *Bridge* Algorithm

**Fig. 2.**
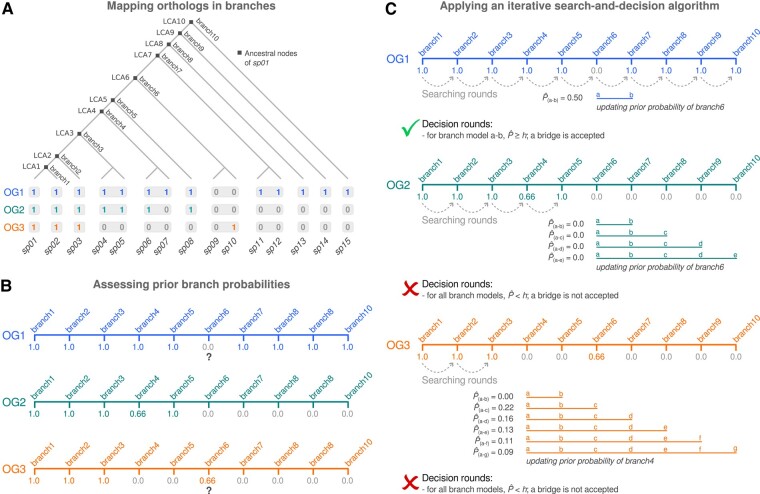
A schematic representation of the *bridge* algorithm. a) A binary matrix with “presences” and “absences” of orthologs obtained for the 3 hypothetical OGs depicted in Fig. 1. b) The probability of occurrence of the OG in each branch. For OG2, the resulting phyletic pattern is consistent with the topology of the branches (i.e. the pattern of the presence and absence of the OG within the analyzed set of branches); however, the phyletic patterns of OG1 and OG3 are inconsistent with the topology of the branches (as denoted by question marks). c) A diagram illustrating the *bridge* algorithm's iterative *search-and-decision* strategy, which identifies the OG root that best aligns with the observed distribution of orthologs (see *The Bridge Algorithm* section).

Given an OG that includes a gene of interest from a designated reference species and a phylogenetic tree with uniquely leaf-labeled species, the *bridge* algorithm will map orthologs to the branches that diverge from the ancestral nodes of the reference species in the tree, assigning “presence” (1) if there is at least one ortholog in a species or “absence” (0) otherwise. Figure 2A displays a binary matrix with “presences” and “absences” from the 3 hypothetical OGs depicted in Fig. 1. Here, the reference species is labeled as *sp01*, and the ancestral nodes of *sp01* represent the evolutionary points (i.e. LCAs) where *sp01* and its related species diverged from a common ancestor. Next, the *bridge* algorithm will assess the probability of occurrence of the OG in each branch. The probability *P* of an OG *O* occurring in a branch *B* is given by:


(1) $$P(O|B)=\frac{g\gamma }{g\gamma +\lambda }\;g>0$$

where *γ* is the number of presences and *λ* is the number of absences of the ortholog in the branch. Our model also considers that “presences” and “absences” will have different contributions to the overall outcome. The rationale for this approach is based on the observation that gene losses may be more common and generally less complex than gene gains (Mirkin et al. 2003). To account for this bias, we introduce *g* as a penalty factor, which will assign higher weights to instances of the minority class during the evolutionary reconstruction. Figure 2B displays the calculated *P* for each branch using *g* = 2. The *bridge* algorithm will then assign the occurrence of the OG in a branch, which will require a decision. Given a local threshold *h* (say, *h* = 0.3), the occurrence of the OG is assigned to branches with *P* ≥ *h* (Fig. 2B; *both g and h are parameterized in the package*). Note that the resulting phyletic patterns of OG1 and OG3 are inconsistent with the topology of the branches, as indicated by question marks.

Next, the *bridge* algorithm will use an iterative search-and-decision strategy, which is outlined in Fig. 2C for OG1, OG2, and OG3. The algorithm takes the list of branches as input, sorting the branches in ascending order, here from branches 1 to 10. The algorithm then iterates through each item, starting with the branch that diverges from the closest ancestral node of the reference species in the tree. During the *searching rounds*, the algorithm assesses each branch, one by one (thereafter called items of a list), with the following possible outcomes:

1. If the current item has *P* ≥ *h*, then the phyletic pattern is satisfied up to that point of the loop.
  1.1 If there are more items on the list, then the loop continues to the subsequent item.
  1.2 If the loop has iterated through all items of the list, then the OG root is found at the ancestral node to all items of the list.
2. If the current item has *P* < *h*, then the algorithm triggers a *decision round*, assessing a posterior probability $\hat{P}$ that takes the evidence of the subsequent item of the list into account.
  2.1 If the current item has $\hat{P}$ ≥ *h*, then the posterior probability is accepted as the new prior. Go back to step 1.
  2.2 If the current item has $\hat{P}$ < *h*, then
    2.2.1 If more subsequent items are available, go back to step 2.1 and repeat the process by using the posterior probability as the new prior and incorporating the new evidence.
    2.2.2 If no more subsequent items are available, then the OG root is found at the ancestral node that does not include the common ancestor to the current and all subsequent items of the list.

The *bridge* algorithm uses the posterior probabilities as weights, aggregating the predictions into a weighted average ensemble model. Given the current item *m* and the subsequent items of the ordered list of branches (*i* = *m*, …, *n*), the posterior probability $\hat{P}$ is given by:


(2) $$\hat{P}(O|B,\mathrm{data})=\sum_{i=m}^{n}\;P(O|B_{i},\mathrm{data})\cdot P(B_{i}|\mathrm{data})$$

where *P*(*O*|*B_i_*, data) is the probability of occurrence of an ortholog *O* given a branch *B_i_* and the observed data. This probability is calculated within each branch separately (see Equation (1)). *P*(*B_i_*|data) is the posterior probability of branch *B_i_* given the observed data, which is calculated during the *decision rounds* for the branch model selection.

Whenever a posterior probability is accepted as the new prior (see step 2.1), a “bridge” is included in the evolutionary model, thus reconciling the phyletic pattern up to that point of the loop. This acceptance step effectively bridges the current item of the loop with the next one, allowing the algorithm to explore additional evidence that might root the ortholog at more ancestral nodes of the tree.

## Uncertainty of Root Placement

After placing the OG root at the given ancestral node of the tree, then the *bridge* algorithm proceeds to estimate uncertainty regarding the root placement, which represents the optimal point that splits the probability distribution into 2 branch ensembles: one enriched with the queried OG (i.e. all branches supporting a vertical heritage pattern) and another one with low evidence in favor of the OG's presence (say branch ensembles *M* and *N*, respectively). Given *M* (*i* = 1, …, *m*) and *N* (*i* = *m* + 1, …, *n*), a consistency score *D* is calculated by aggregating these 2 ensembles as follows:


(3) $$M=\sum_{i=1}^{m}\;P(O|B_{i},\mathrm{data})\cdot P(B_{i}|\mathrm{data})$$

(4) $$N=\sum_{i=m+1}^{n}\;P(O|B_{i},\mathrm{data})\cdot P(B_{i}|\mathrm{data})$$

(5) D=M−N

where *m* is the root position in the ordered list of branches, and *n* is the last branch of the ordered list. The score *D* will be 1 when the vertical heritage pattern exhibits maximal consistency, that is, *M* = 1 and *N* = 0. As a final step, the *bridge* algorithm calculates an empirical *P*-value associated with the *D* score using permutation analysis, randomly shuffling the data points to create a null distribution. The algorithm typically repeats the permutation process a large number of times (e.g. 1,000 or more) and then compares the observed and null scores to determine the probability of obtaining a *D* score as extreme or more than the observed one by chance. In the following section, we simulate random OGs to benchmark the algorithm against different phyletic patterns, demonstrating the distribution of *D* scores and associated uncertainty levels.

## Benchmarking Accuracy

**Fig. 3.**
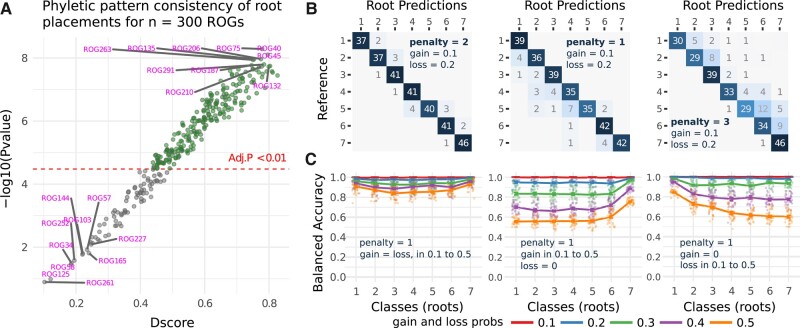
The performance of the *bridge* algorithm to predict roots for 300 simulated random ROGs. a) An uncertainty of root placement. Each data point displays a consistency score (*D*) along with the associated *P*-value (on a −log10 scale) calculated for a simulated ROG. A low *P*-value (green circles) indicates that the predicted root is supported by a phyletic pattern in the data. Each ROG was simulated using default parameters of the *simulateRogs*() function available in the *GeneBridge* package. b) Three confusion matrices showing ROG's roots predicted by the *bridge* algorithm for varying penalty factors. The ROG simulation generated reference roots by modeling ideal vertical heritage patterns. These patterns were then subjected to random gain and loss events at fixed probability levels. The simulation used a random phylogenetic tree with 7 possible roots, referred to as classes. c) Performance plots depicting balanced accuracy across the different root classes, using a fixed penalty factor in the *bridge* algorithm, while varying gain and loss probabilities in the ROG simulation. A detailed reproduction of the simulation is provided in the vignette of the *GeneBridge* package.

Benchmarking the accuracy of a new algorithm poses a nuanced challenge. The complexity arises from the need for a well-defined standard against which the accuracy of the evaluated method can be measured. To assess the accuracy of root assignments, we simulated random OGs (ROGs) by modeling gain and loss events within a given phylogenetic tree. The simulation starts by assigning a random root to each ROG. Following that, a vertical heritage is propagated through the tree, extending up to a designated reference species. The resulting structure is then subjected to random gain and loss events. The primary goal of the simulation is to generate ROGs exhibiting diverse phyletic patterns, deviating from an exact vertical heritage based on the level of random events applied in the simulation. A detailed reproduction of the simulation is provided in the vignette of the *GeneBridge* package. Figure 3A displays the distribution of *D* scores for 300 ROGs. The *P*-value represents a measure of the evidence against the null hypothesis, which here asserts that the inferred root is not consistent with a vertical heritage pattern. A low *P*-value (e.g. green circles) would suggest that the observed data are unlikely to occur if the inheritance pattern is truly not vertical, and a high *P*-value (e.g. gray circles) indicates a low conformity of root placements with a vertical heritage pattern.

Next, we simulated reference roots under different parametrizations using a random phylogenetic tree with 7 possible roots (referred to as classes here), and then we examined the predicted roots obtained by the *bridge* algorithm. Figure 3B illustrates 3 confusion matrices obtained by varying the penalty factor, which should be used to adjust the *bridge* algorithm based on the expected probability of gain and loss events. When penalty = 1, equal probability is expected; penalty > 1 indicates a higher probability of gene loss, while penalty < 1 indicates a higher probability of gene gain. For the left confusion matrix, we assigned penalty = 2, gain = 0.1, and loss = 0.2, representing the default parametrization. Of the 300 ROGs, only 17 were inaccurately predicted by the *bridge* algorithm (i.e. not placed on the diagonal). Modifying the penalty factor for the same proportion of gain and loss events will result in less accurate predictions, as reflected in the other 2 confusion matrices (center and right). Figure 3C depicts the balanced accuracy for a fixed penalty factor, but varying gain and loss events. This shows that the *bridge* algorithm is responsive to the random events that potentially disrupt the phyletic pattern. Optimal accuracy is achieved when the penalty factor is appropriately set to align with the expected proportion of gain and loss events (left accuracy plot). It is worth noting that the root classes at the extreme ends exhibit a higher accuracy, underscoring the interplay between gain and loss events, which have opposite effects in disrupting the phyletic pattern (center and right accuracy plots).

## Implementation

The *bridge* algorithm is implemented in *R* (R Core Team 2021) and is available in the *GeneBridge* package, which runs on Linux, MacOS, and Windows platforms. The software's data input consists of an OG dataset and a phylogenetic tree. The OG dataset can be obtained from orthology databases, while the phylogenetic tree should contain the evolutionary relationships between all species annotated in the OG dataset. We also provide preprocessed data from STRING (Szklarczyk et al. 2019), OrthoDB (Kriventseva et al. 2019), and OMA Browser (Altenhoff et al. 2021) databases for use in the *GeneBridge* package, including a function that allows users to parse OGs from these orthology databases into a tabular format, ready to be used in the *GeneBridge* package. The *bridge* algorithm has been implemented taking advantage of its use for an exploratory data analysis of different genetic systems (Oliveira et al. 2019; Trefflich et al. 2020; de Souza et al. 2021; Viscardi et al. 2021), each of these studies demonstrating the usability of the framework to explore network evolutionary scenarios. The *GeneBridge* package offers extensive documentation that showcases the analysis workflow, including instructions on how to customize the user's input data. For additional details, refer to the package's vignettes available in the github repository (*GeneBridge* version ≥0.99.1).

## Performance, Strengths, and Limitations

We assessed the performance of the *bridge* algorithm for evaluating OG datasets of increasing sizes (supplementary fig. S1, Supplementary Material online). The *bridge* algorithm's runtime grows proportionally with the input size, indicating that the algorithm's performance scales well for large datasets. It is important to highlight that the *bridge* algorithm does not aim to explain all possible evolutionary scenarios of a given OG. Instead, its primary objective is to identify the OG root that best aligns with the observed distribution of orthologs across the branches that diverge from the ancestral nodes of the reference species. In this context, when analyzing the inferred OG root, the interpretation should be confined to the genes of the reference species and ideally representing a relative evolutionary distance between those genes. Therefore, the *bridge* algorithm comes with a trade-off of not resolving more granular scenarios, such as the reconstruction of OGs at all taxonomic levels of the species tree.

## Supplementary Material

msae019_Supplementary_Data

## Data Availability

The data underlying this article are available at https://github.com/sysbiolab/GeneBridge.
