# Supplementary figures and images for: Bridge: A New Algorithm for Rooting Orthologous Genes in Large-Scale Evolutionary Analyses
Source: Mol Biol Evol. 2024 Feb 2;41(2):msae019. doi: 10.1093/molbev/msae019 (PMC10873778; doi:10.1093/molbev/msae019)

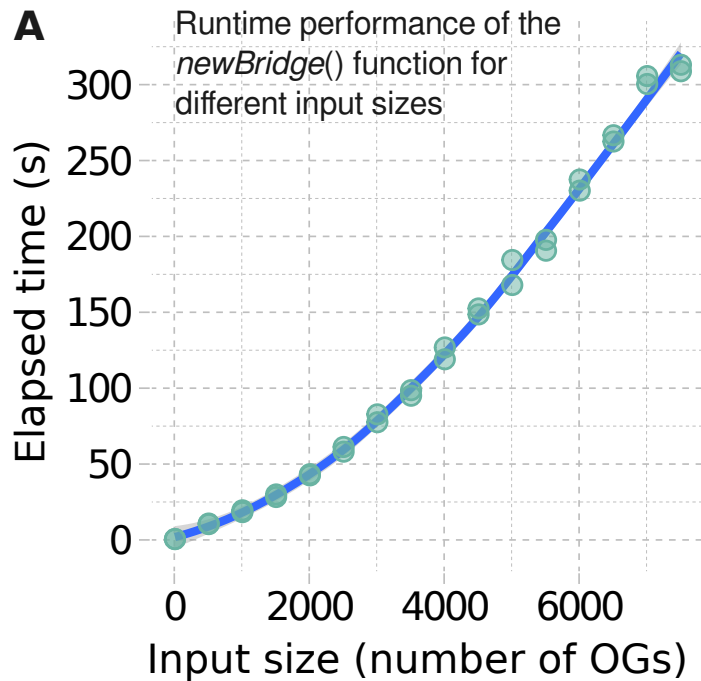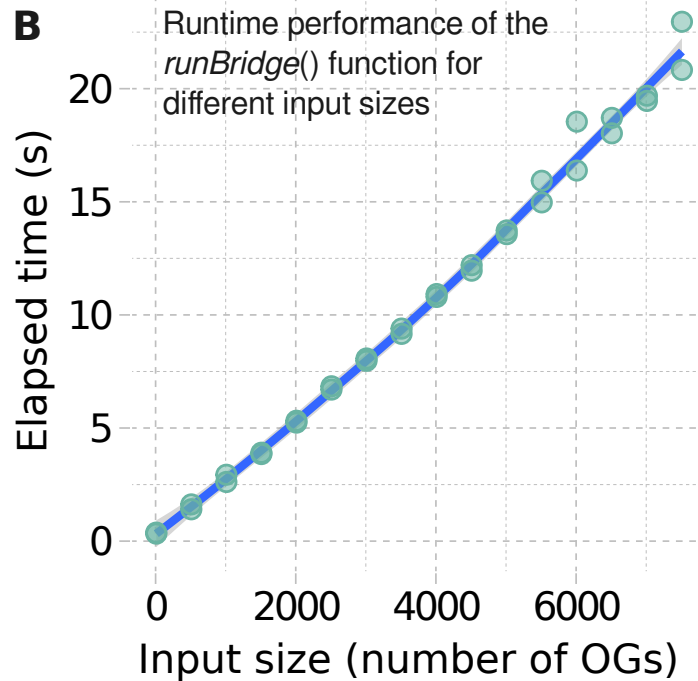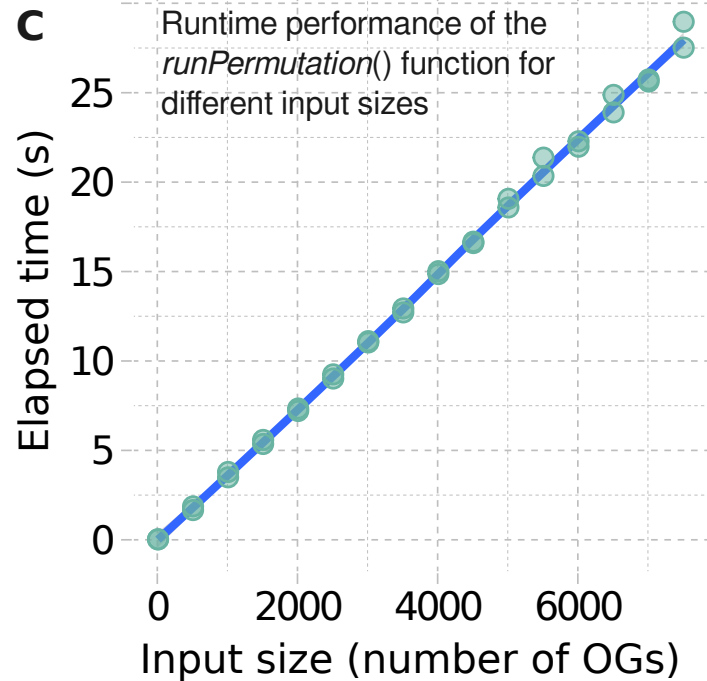

Supplement: msae019_Supplementary_Data [file msae019_supplementary_data.pdf]
